# Burnout among medical students during the first years of undergraduate school: Prevalence and associated factors

**DOI:** 10.1371/journal.pone.0191746

**Published:** 2018-03-07

**Authors:** Robson Aparecido dos Santos Boni, Carlos Eduardo Paiva, Marco Antonio de Oliveira, Giancarlo Lucchetti, José Humberto Tavares Guerreiro Fregnani, Bianca Sakamoto Ribeiro Paiva

**Affiliations:** 1 Barretos School of Health Sciences, Dr. Paulo Prata, Barretos, São Paulo, Brazil; 2 Research Group on Palliative Care and Health-Related Quality of Life (GPQual) –Barretos Cancer Hospital, Barretos, São Paulo, Brazil; 3 Teaching and Research Institute, Barretos Cancer Hospital, Barretos, São Paulo, Brazil; 4 Department of Clinical Oncology – Breast and Gynecology Division, Barretos Cancer Hospital, Barretos, São Paulo, Brazil; 5 School of Medicine, Federal University of Juiz de Fora, Minas Gerais, Brazil; Universidade de Mogi das Cruzes, BRAZIL

## Abstract

**Objective:**

To evaluate the prevalence and possible factors associated with the development of burnout among medical students in the first years of undergraduate school.

**Method:**

A cross-sectional study was conducted at the Barretos School of Health Sciences, Dr. Paulo Prata. A total of 330 students in the first four years of medical undergraduate school were invited to participate in responding to the sociodemographic and Maslach Burnout Inventory-Student Survey (MBI-SS) questionnaires. The first-year group consisted of 150 students, followed by the second-, third-, and fourth-year groups, with 60 students each.

**Results:**

Data from 265 students who answered at least the sociodemographic questionnaire and the MBI-SS were analyzed (response rate = 80.3%). One (n = 1, 0.3%) potential participant viewed the Informed Consent Form but did not agree to participate in the study. A total of 187 students (187/265, 70.6%) presented high levels of emotional exhaustion, 140 (140/265, 52.8%) had high cynicism, and 129 (129/265, 48.7%) had low academic efficacy. The two-dimensional criterion indicated that 119 (44.9%) students experienced burnout. Based on the three-dimensional criterion, 70 students (26.4%) presented with burnout. The year with the highest frequency of affected students for both criteria was the first year (p = 0.001). Personal attributes were able to explain 11% (ΔR = 0.11) of the variability of burnout under the two-dimensional criterion and 14.4% (R^2^ = 0.144) under the three-dimensional criterion.

**Conclusion:**

This study showed a high prevalence of burnout among medical students in a private school using active teaching methodologies. In the first years of graduation, students’ personal attributes (optimism and self-perception of health) and school attributes (motivation and routine of the exhaustive study) were associated with higher levels of burnout. These findings reinforce the need to establish preventive measures focused on the personal attributes of first-year students, providing better performance, motivation, optimism, and empathy in the subsequent stages of the course.

## Background

The mental health of medical students has been a cause for concern on the part of medical schools in several countries: in addition to their study-related burdens, many demands and responsibilities are placed on these students because the profession is dedicated to the health care of people and has very little tolerance for mistakes, favoring the development of stress and anxiety [1–3]. Burnout is a multifactorial occupational syndrome, characterized by a triad of symptoms involving high levels of emotional exhaustion, depersonalization, or professional cynicism and professional disbelief that are prominent in early manifestations, involving especially medical students [4]. Studies have shown that having at least one burnout symptom can cause negative effects in medical students that not only interfere with the teaching/learning process but also cause drowsiness, fatigue, eating disorders, migraine, emotional instability, and even the use of illicit drugs [5,6]. Based on these aspects and the fact that burnout has already been reported in different professions, there is a substantial prevalence of this syndrome among medical students. On average, in the first four years of medical school, 34% of the students exhibit moderate levels of burnout; in Brazil, the average prevalence is 65.1% [7–9]. An inducer of burnout in students is associated with the pedagogical project of the medical course. A growing number of medical schools and postgraduate courses are adopting active methodologies as teaching/learning instruments, with the goal of updating the teacher-centered pedagogical format practiced for centuries [10]. The active methodology of teaching can cause anxiety in students [10]. It is known that students who enter medical schools whose teaching methodologies differ from those experienced in elementary education have a great chance of developing stress and anxiety compared with students who enter schools with conventional teaching methods [11]. However, when comparing two Brazilian medical schools with different teaching methodologies, Botelho et al. [11] perceived that stress levels were high in both schools, with prevalence rates above 65%. With the evolution of the active course, better adaptation and understanding of the teaching method were observed, leveraged by the factors of proximity with the facilitator professor, the use of real clinical cases, study in small groups, and the help of a tutor in the organization and orientation of the learning, but without any perception of improvement of the symptoms of burnout [10]. Some specific student-centered teaching strategies, such as problem-based learning (PBL), can also confer high levels of stress and distress to students, often due to doubts about the consistency of the students’ training and apprehension due to the process of evaluation of the content learned; a prevalent feeling among these students is unpreparedness [7,12]. In schools with traditional, teacher-centered teaching methods, stress levels among students are strongly associated with the medical training process, with the workload and the sense of oppression imposed by the routine of studies being the major causes of stressor events [12]. Furthermore, academic pressure, sleep deprivation, social and family expectations, financial difficulties, and daily contact with patient suffering and death catalyze the psychological morbidity experienced by students, with depressive symptoms and deteriorating mental health [13].

Regarding the symptoms of burnout for non-medical professionals, the manifestations are triggered by overwork, unfavorable conditions, job insecurity, and instability [13]. However, studies that combine the use of an inventory, such as the Maslach Burnout Inventory—Student Survey (MBI-SS), with other specific variables linked to the daily life of medical students to determine which students are more prone to burnout symptoms are still scarce. Among professionally active physicians, Gracino et al. [14] postulated that the dimensions of burnout syndrome manifest themselves sequentially, developing emotional exhaustion first, followed by depersonalization in the attempt to deal with exhaustion; finally, the ability to resist the demands of work diminishes, resulting in feelings of anguish and low professional productivity. According to the NEJM report and members of the Catalyst Insights Council, burnout is a growing problem and difficult to solve; experts have cited that approximately ¼ of physicians have burnout [15]. In a Brazilian study that included an analysis of the literature between 2006 and 2015, 12.1% of physicians and 10.6% of students in general had burnout. An important finding is that of individuals with burnout, 54.6% were young adults [9].

With the possible damages caused by burnout in mind, a meta-analysis of interventions to prevent and reduce physician burnout conducted by West et al. [16] identified some important strategies able to reduce burnout symptoms, such as the involvement of mindfulness, stress management, and duty hour limitation policies.

With this aim, this study sought to identify and quantify the importance of possible factors causing burnout among students in the first years of an undergraduate medical school that uses the active methodology.

## Methods

### Study design, setting, and ethical issues

This was an observational cross-sectional study conducted at the Barretos School of Health Sciences, Dr. Paulo Prata (Barretos, São Paulo state, Brazil) from June 2015 to June 2016.

The Barretos School of Health Sciences, Dr. Paulo Prata is a private college founded in 2012 and is divided into four years of pre-clinical and clinical content and two years of clerkship. The teaching methodology follows the active student-centered model called PBL or team-based learning (TBL) and involves simulations with dynamic activities designed to stimulate integrated reasoning and expansion and conceptual sedimentation of previous knowledge.

The study was performed in accordance with the international ethical standards of the Declaration of Helsinki and the Brazilian National Health Council Resolution no. 466/2012 and was approved by the Ethics Committee of the Barretos Cancer Hospital (HCB no. 1.130.726/2015 and CAAE 45741115.2.00005437). Volunteers indicated their agreement to participate in the study via the electronic informed consent included in the survey form.

Students identified as having probable burnout were individually notified by the investigators in accordance with the signed consent form and were referred to the Barretos School of Health Sciences teaching support center. The center is composed of a psychiatrist, a psychologist, and a pedagogue.

### Eligibility criteria

Students included in the study were those enrolled in the first four years of the Barretos School of Health Sciences, Dr. Paulo Prata undergraduate medical course who voluntarily agreed to participate by signing the consent form.

### Procedures

First, a lecture was given to medical students about "burnout", informing them about the conduct of the research. Then, a message was sent (via a messaging application) containing the link to the data collector, allowing access to the informed consent form, along with the assessment of the socio-demographic issues of daily life and the MBI-SS. Data collection occurred outside the curricular evaluation period, and the students attended routine school activities (theoretical activities of directed study and laboratory and outpatient practices). To facilitate the completion of the questionnaires and increase the guarantee of confidentiality of the data, we chose to collect online questionnaire responses using the *SurveyMonkey*^®^ program, which was acquired legally from the registration on the site (https://pt.surveymonkey.com).

### Instruments

The questionnaire used took an average of 25 minutes to complete and contained the following instruments:

- Evaluation of sociodemographic data: variables such as sex, marital status, undergraduate school year, number of children, family income, work activities not associated with medicine, and religion.
- Questionnaire regarding variables related to the daily life of medical students: the authors of the research had meetings and developed a questionnaire based on the scientific literature on medical education. The purpose of this questionnaire was to identify key aspects of life for which it was possible to gather information about the students’ daily routines related to family life, religiosity/spirituality, academic performance, interpersonal relationships, and sports and leisure. The authors classified the questions into three categories: school domain, personal domain, and outside-of-school domain. This questionnaire was composed of 18 questions with Likert type responses; for example, *Do you feel happy as a medical student*? (*Not at all—Slightly—Somewhat—Very—Extremely*) or questions with affirmative or negative answers; for example, *Do you currently feel fulfilled as a medical student*? *(yes or no)*. The questions were classified into domains based on the students’ relationships with their respective daily activities, and the answers to the questions were dependent on the domains. For example: Personal domain—*Do you consider yourself exhausted*?; school domain: *Your current study routine is…*; outside-of-school domain—*How often do you and your family meet for a family reunion at home*?
- Burnout assessment: This evaluation was performed through the MBI-SS instrument, which corresponds to an instrument containing 15 unique items for the evaluation of burnout in students, not taking into account the antecedent elements and the consequences resulting from their process. The MBI-SS is divided into three distinct domains: emotional exhaustion (low = 0–9; moderate = 10–14; high > 14), cynicism (low = 0–1; moderate = 2–6; high > 6), and academic efficacy (low ≤ 22; moderate = 23–27; high ≥ 28) [17]. Elevated scores for emotional exhaustion and cynicism and low scores for academic efficacy indicate high levels of burnout [18]. The two-dimensional criteria (high scores for emotional exhaustion and cynicism) and three-dimensional criteria (high scores for emotional exhaustion and cynicism and low scores for academic efficacy) were used as the criteria for the diagnosis of burnout [17]. The validated survey version in Portuguese/Brazil presented adequate psychometric properties[18,19]. In the present study, the Cronbach’s alpha values were 0.829 (emotional exhaustion), 0.741 (cynicism), and 0.803 (academic efficacy), and the confidence interval (CI) used was a 95% CI. The MBI-SS has had its right of use paid and has been duly authorized by *Mind Garden*, as described on the website http://www.mindgarden.com/.

### Sample size

All the students enrolled in the first to the fourth year of medical school were invited to participate in the study via the Informed Consent Form (ICF). Therefore, this sample represents a convenience sample.

### Data analysis

The study population was characterized using frequency tables for qualitative variables and a mean and standard deviation for the age of the participants. The scores were described after categorization using a frequency table. The χ^2^ test was used to compare the scores according to the year of undergraduate school and the personal, educational, and outside-of-school characteristics of the students. Those comparisons with a p-value less than 0.2 were selected for the multivariate analysis. A *post-hoc* analysis was performed using the χ^2^ test at the corrected significance level based on the Bonferroni method. Multiple logistic regression was used, in which the independent variables were divided into four blocks (year of undergraduate school, personal, educational, outside-of-school). The initial model was adjusted only for the year of undergraduate school, and in each subsequent model, a block of variables was added using the stepwise-forward method to select the variables within each block. Thus, the final model was composed only of the characteristics that were jointly significant (p < 0.05). The *SPSS Statistics version 21*.*0 for Windows* software from IBM was used for the analysis. The level of statistical significance adopted was 5% (p < 0.05).

## Results

### Sample description

Of a total of 330 medical students enrolled in the first to the fourth years, 50 (12.06%) did not open the link to the data collector, one student (0.4%) read the ICF and did not agree to participate in the research, and 279 (81.81%) answered only the sociodemographic questionnaire (Table 1). Of the 279 students, 14 (5.01%) did not fully answer the MBI-SS questionnaire. Thus, 265 respondents were analyzed regarding this questionnaire.

**Table 1:** Sociodemographic characterization of medical students at the Barretos School of Health Sciences, Dr. Paulo Prata, Brazil (n = 279).

Variables	
**Gender (n, %)**	
Female	183 (66.1%)
Male	96 (33.9%)
**Age (mean, SD**^a^**)**	21.4 (2.7)
**Marital Status (n, %)**	
Married	5 (1.8%)
Separated/Divorced	2 (0.7%)
Single	265 (94.9%)
Other	7 (2.5%)
**Stage of course (n, %)**	
First Year (first and second periods)	118 (42.6%)
Second Year (third and fourth periods)	59 (21.3%)
Third Year (fifth and sixth periods)	51 (18.4%)
Fourth Year (seventh and eighth periods)	49 (17.7%)
**Ethnicity/Race**^b^ **(n, %)**	
Black	2 (0.7%)
White	265 (95.6%)
Asian	8 (2.8%)
Brown	2 (0.7%)
**Children (n, %)**	
Yes	1 (0.4%)
No	278 (99.6%)
**Total Family income (dollars)**^c^ **(n, %)**	
Less than $1,500	38 (12.7%)
$1,500 to $3,000	107 (38.8%)
$3,000 to $5,000	61 (22.1%)
$5,000 to 6,500	25 (9.1%)
+ from $6,500	48 (17.4%)
**Work (n, %)**	
Yes	5 (1.8%)
No	274 (98.2%)
**Religious affiliation (n, %)**	
Catholic	157 (49.8%)
Spiritist	46 (16.4%)
Evangelical	21 (7.5%)
**I have no formal religion**	**55 (19.8%)**

^**a**^ SD = standard deviation.

^B^ Santos et al.[20].

^**C**^ The Brazilian currency (Brazilian Real) converted into US dollars, using the average exchange rate for the period (R$3.25).

### Prevalence of burnout syndrome

Burnout scores were categorized in terms of the emotional exhaustion, cynicism, and academic efficacy domains. Based on this categorization, 187 (n = 187, 70.6%) students were classified as having high emotional exhaustion, 140 (n = 140, 52.8%) as having high cynicism, and 129 (n = 129, 48.7%) as exhibiting low academic efficacy. According to the two-dimensional criterion (high exhaustion + high cynicism), 119 (n = 119, 44.9%) students were experiencing burnout, as shown in Table 2.

**Table 2:** Prevalence of burnout syndrome among medical students according to the subscales of the *Maslach Burnout Inventory Student Survey* (MBI-SS) (n = 265).

Categorized Domains (RV)	N (%)
**MBI Emotional Exhaustion**	
Low (0–9)	23 (8.7)
Moderate (10–14)	55 (20.8)
High (> 14)	187 (70.6)
**MBI Cynicism**	
Low (0–1)	23 (8.7)
Moderate (2–6)	102 (38.5)
High (> 6)	140 (52.8)
**MBI Academic Efficacy**	
Low (≤ 22)	129 (48.7)
Moderate (23–27)	78 (29.4)
High (≥ 28)	58 (21.9)
**Two-dimensional burnout** ^1^	
No	146 (55.1)
Yes	119 (44.9)

^**1**^High emotional exhaustion + high cynicism.

^**2**^High emotional exhaustion + high cynicism + low scores on the **academic** efficacy subscale.

**RV** = Reference Values.

### Univariate analysis

#### Characteristics associated with burnout

The burnout scores of the medical students were different according to the year of undergraduate school (p = 0.001); scores were higher among students in the first year relative to students in the other years (Table 3).

**Table 3:** Association between two-dimensional burnout diagnosis and undergraduate school year (Barretos School of Health Sciences, Dr. Paulo Prata, 2015–2016) (n = 265).

Variables	*Burnout*		
	No N (%)	Yes N (%)	p*
***Undergraduate school year***			0.001
First Year	46^**a**^ (40.7)	67^**b**^ (59.3)	
Second Year	37^**a**^ (66.1)	19^**a**^ (33.9)	
Third Year	34^**a**^ (69.4)	15^**b**^ (30.6)	
Fourth Year	29^**a**^ (61.7)	18^**a**^ (38.3)	

*p-value *X*^*2*^ test with linear tendency.

each letter (a and b) denotes a subset of two-dimensional and three-dimensional categories of burnout with column proportions that do not differ significantly from each other.

Among the variables of the personal domain, poor self-perception of health (p < 0.001), not being optimistic (p = 0.04), and not feeling fulfilled as a medical student (p = 0.002) were associated with increased levels of burnout. For school characteristics, a long time spent in college (p < 0.001), feeling worn out and dissatisfied as a student (p = 0.03), and a demotivation to study (p < 0.001) had a significant association with burnout for the two-dimensional criterion. Among the outside-of-school characteristics, a low frequency of family encounters (p < 0.001), lack of leisure time (p = 0.005), and low physical activity (p = 0.03) were significantly associated with burnout (Table 4).

**Table 4:** Association between two-dimensional scores and the variables of the personal, school, and outside-of-school domains of medical students (Barretos School of Health Sciences, Dr. Paulo Prata, 2015–2016) (n = 265).

Variables	With Burnout N (%)	p**
***Personal Domain***		
***Gender***		0.509
Female	82 (68.9)	
Male	37 (31.1)	
Family income (*)		0.433
≤ $3,000.00	57 (47.9)	
≥ $3,000.00	62 (52.1)	
***Self-perception of health***		**< 0.001**
Bad	57 (47.9)	
Good	62 (52.1)	
***Any health problems***		0.133
Yes	33 (27.7)	
No	86 (72.3)	
***Optimism***		**0.043**
Not optimistic	79 (66.4)	
Optimistic	40 (33.6)	
***Financial satisfaction***		0.758
Dissatisfied	33 (27.7)	
Satisfied	86 (72.3)	
***Fulfillment as a medical student***		**0.002**
No	35 (29.4)	
Yes	84 (70.6)	
***School Domain***		
***School activities***		0.057
Undergraduate school + other activity	90 (75.6)	
Undergraduate student only	29 (24.4)	
***Average hours in college/day***		**< 0.001**
Up to 12 hours	99 (83.2)	
More than 12 hours	20 (16.8)	
***Feeling worn out or dissatisfaction as a student***		**0.038**
Yes	118 (99.2)	
No	1 (0.8)	
***Motivation for studies***		**< 0.001**
No	54 (57.4)	
Yes	40 (42.6)	
***Routine of studies***		**0.013**
Exhaustive	89 (74.8)	
Not exhaustive	30 (25.2)	
***Consider yourself important to colleagues***		0.176
No	47 (39.5)	
Yes	72 (60.5)	
***Satisfaction as a student***		0.067
Dissatisfied	64 (53.8)	
Satisfied	55 (46.2)	
***Outside-of-school Domain***		
***Frequent family meetings***		**0.021**
No	81 (68.1)	
Yes	38 (31.9)	
***Leisure time***		**0.005**
No	110 (92.4)	
Yes	9 (7.6)	
***Has religion***		0.324
No	27 (22.7)	
Yes	92 (77.3)	
***Influence of religious/spiritual life in studies***		0.443
No	78 (65.5)	
Yes	41 (34.5)	
***Physical activity***		**0.030**
No	44 (37.0)	
Yes	75 (63.0)	
***Consider yourself important to your family members***		0.837
No	2 (1.7)	
Yes	117 (98.3)	

* value (Brazilian currency) converted into dollars.

** p-value Mann-Whitney test.

### Multivariate analysis

The logistic regression model was initially adjusted for the year of undergraduate school, and a block of variables was added at each stage of analysis. Undergraduate school year alone was able to explain only 8% of the variability of the burnout frequency (R^2^_adjusted_ = 0.08). The personal and school attributes were able to explain 11% (ΔR^2^ = 0.11) and 6% (ΔR^2^ = 0.06) of the variability, respectively. In the end, Model 3 was able to explain 25% of the burnout frequency variability. The final model (Model 3) showed that students with a good self-perception of health (OR = 0.3, p = 0.003), who were optimistic (OR = 0.5, p = 0.037), who were motivated to study (OR = 0.4, p = 0.006), and who had a non-exhaustive study routine (OR = 0.5, p = 0.033) had lower chances of developing burnout (Table 5).

**Table 5:** Multiple logistic regression model of the two-dimensional criterion for burnout adjusted for the year of undergraduate school and the personal and educational attributes of medical students (Barretos School of Health Sciences, Dr. Paulo Prata, 2015–2016) (n = 265).

Variables	Model 1		Model 2		Model 3	
	OR (95% CI)	p*	OR (95% CI)	p*	OR (95% CI)	p*
**Block 1—Undergraduate school year**						
First year	Ref 1.0	-	Ref 1.0	-	Ref 1.0	-
Second year	0.3 (0.1–0.6)	**0.002**	0.4 (0.2–0.8)	0.096	0.6 (0.2–1.4)	0.266
Third year	0.3 (0.1–0.6)	**0.001**	0.3 (0.1–0.8)	0.085	0.5 (0.2–1.1)	0.109
Fourth year	0.4 (0.2–0.8)	**0.017**	0.5 (0.2–1.1)	0.357	0.8 (0.3–1.9)	0.670
**Block 2—Personal attributes**						
Self-perception of health (good/bad)			0.2 (0.1–0.5)	**< 0.001**	0.3 (0.8–0.7)	**0.003**
Optimism (no/yes)			0.5 (0.3–1.0)	**0.025**	0.5 (0.2–0.9)	**0.037**
**Block 3—School attributes**						
Motivation in studies (no/yes)					0.4 (0.2–0.7)	**0.006**
Exhausting study routine (no/yes)					0.5 (0.2–0.9)	**0.033**
**R**^**2**^ **adjusted**	**0.08**		**0.19**		**0.25**	
**Change in R**^**2**^			**0.11**		**0.06**	

**OR** = Odds ratio.

*p-value obtained by Multiple logistic regression.

Further analyses were conducted considering the three-dimensional criteria for burnout (please see S1, S2, S3 and S4 Tables).

## Discussion

In this study, we investigated potential factors of the daily life of medical students that might be associated with the development or severity of burnout in a private college with a curriculum based on student-centered teaching methodology and simulations. We found that personal factors were the most relevant to the development of burnout in medical students, followed by the students’ training phase (year of course) and conditions related to studies.

Regarding the levels of burnout, we observed that more than 70% of participants had high levels of emotional exhaustion and approximately half of the students presented with cynicism, leading to a burnout prevalence of 44.9%. These results are similar to those from other studies conducted internationally, in which the burnout prevalence on average ranges from 21 to 71%[8,13,21], and are also similar to Brazilian studies that present mean prevalence values of 14.9 to 65.1%[1,22]. High levels of burnout in medical school are a result of the increasing degree of difficulty imposed on the students by the courses, the frequent contact with sick people and death, and the routinely experienced conflict between the rational and the emotional[12,22,23].

A systematic review by Ishak et al.[21] revealed that the main negative personal factors associated with increased burnout rates were experiences with serious illness. The authors also noted that student exhaustion due to a low-rest routine was a predictor of burnout for first- and second-year students, while third- and fourth-year students suffered from organizational difficulties and sleep deprivation.

In this study, we identified that aspects of health, optimism, and achievement as a student were the factors most associated with burnout. Dyrbye et al.[24] reported that stressors in an individual’s personal and professional life can cause high levels of stress, depression, anxiety, and burnout. Our findings on personal factors, when interpreted and correlated, agree with the literature.

The existence of an association among personal characteristics, an affirmative personality, and empathy favors building relationships, which can be "positive attributes" as opposed to "negative attributes", which are usually detrimental to interpersonal relationships. These notes by Hojat et al.[25] indicated that students with good personal characteristics related to personal fulfillment experience protective effects against the development of burnout. The same protective effects are also conferred by optimism, which elevates personal fulfillment and reduces emotional exhaustion [15].

The outside-of-school environment, especially as it involves family problems, is believed to have a small influence on the development of burnout [16,24], first, because burnout is a syndrome related to a triad of factors and, second, because the great majority of authors describe family closeness as a protective factor that lowers the threshold of stress, lowers distress, and increases happiness. These characteristics are reinforced by the fact that, when out of school, stressors induced by intense study, assessment periods, and contact with the death of patients are not present [8,26,27].

Students entering the undergraduate medical course had higher levels of burnout [28,29]. These high levels of burnout among freshmen are worrisome since the syndrome has been described among active professionals, and this finding can be perceived as an increasingly early incidence of burnout[22]. In fact, students have a high level of stress in stages prior to admission to medical school because many of them spend several years trying to be admitted to medical school without success, which considerably increases the expectations imposed by society and the family[30]. It is understandable that the joy and personal relief when entering college is momentary, as this joy is soon replaced by an intense study routine, time and personal financial management, and distance from the family[31,32]. The management of everyday life is usually complex for these young students. In addition, there is a spirit of competitiveness, frequent frustrations, tensions in the university and hospital environment, observations of the exhaustive practices of medical professionals, and a lack of leisure and rest[30,33,34]. All of these factors may have been responsible for the high levels of burnout observed in the first year of the course, which are compatible with the results of other Brazilian studies that showed worse mental health and burnout in medical students from the first periods compared with other periods[32,35].

Heinen et al.[7] showed that first-year students of the medical course experience high levels of stress and compromised optimism. These findings deserve attention, as they reinforce the idea that students begin the medical course with traces of emotional and affective impairment.

Another factor highlighted by the present study, despite having less of an association with burnout than the other factors, was the school attribute, mainly the motivation and routine of studies. Studies show that these motivational factors lead to greater school engagement, enhanced empathy with patients, good interpersonal relationships, satisfactory performance in school activities, and better emotional control [36]. Santen et al. [8] described a variation found in the literature, where several authors use only the one-dimensional (emotional exhaustion) or two-dimensional (emotional exhaustion and cynicism) criterion to classify burnout, although the original scale uses the three-dimensional criterion. The feeling of professional effectiveness is relevant since the last attribute, when present in a positive way, behaves as a protective factor to decrease the level of burnout. Another important protective factor for students, as shown in the literature, is empathy; empathic students are more personally accomplished and perform better [2,8,27,37]. These characteristics may contribute to the reduction of burnout levels from the second year of graduation, but due to the multifactorial characteristics of burnout syndrome, students’ behavior may suffer positive or negative variations due to the variability of the personal and school context [24,38,29,39].

The curriculum itself, the pedagogical format, and the learning environment of medical schools are also associated with the perception of burnout[40].

One study showed that active teaching strategies are associated with high burnout rates compared with other methods [41]. The main reason for this higher burnout rate is the fact that the students did not know what the faculty expected of them. There were too many small group sessions facilitated only by students, resulting in an unclear curriculum. There was also a lack of opportunity to explore academic subjects of interest. Another study also showed that students of courses run predominantly using the PBL method studied under conditions of great stress due to doubts about the consistency of their training and the apprehension generated by the assessment process of the learned content. These factors led to feelings of unpreparedness [12]. These data are still preliminary, and it is not known how much of the problem involves the strategy used, in this case PBL, and how much involves the way in which this strategy is used, especially since systematic reviews point to better student knowledge and satisfaction among those taught by this method[42–44]. Our findings, in agreement with the literature[8], provide evidence that burnout is a chronic health condition with little prospect for improvement as the student continues in the medical course. Students who perceive or present with some illness or who are unmotivated or deprived of regular nights of sleep due to their exhaustive routine are more likely to be those who present high levels of burnout[45].

The primary goal of any method of medical education is to train medical professionals with broad biological and psychosocial knowledge and technical skills[24]. Thus, students with high levels of stress and burnout for long periods without intervention may have substantially impaired professional training, be exposed to illicit drugs, abuse alcohol, or experience mental disorders and suicidal tendencies[2,13]. They are also more prone to medical errors, unpreparedness, and lack of empathy[7,41].

To minimize these negative repercussions in the training of future physicians, protective strategies have been proposed in the literature, such as physical activity, adequate sleep, psycho-pedagogical support, educational strategies, and a better learning environment[7,24]. Therefore, strategies to prevent or treat students with burnout, through curricular flexibility or psycho-pedagogical management to better understand each student’s profile, are promising ways to help reduce burnout among students. These strategies may also include reducing the weight of daily school activities and introducing wellness programs and moments of meditation [29]. Educators and management policies can create methods to increase the confidence and personal motivation of students with the fundamental purpose of increasing empathy and enjoyment in study, thus fostering a healthy well-being as a strong personal factor of protection [36].

### Limitations of the study

This study has some limitations. First, the study was conducted in a single private medical school with active learning methodology, which diminished the generalization power of the study. Although the instrument was carefully prepared by the authors and was based on the literature, the questionnaire to evaluate the daily activities of medical students was developed by the study authors and was not previously validated, which could be considered another limitation. Within the construction of personal variables, an assessment of personality measures would be interesting. Another issue was the need to limit the number of questions to obtain as many answers as possible. This study compared students who presented burnout from different school years, but a longitudinal assessment of these students would be a more appropriate approach. The lack of a group of non-medical students as a control and the fact of closeness among students who did not participate in the study is also a limiting factor since the strong comorbidities of depression, anxiety, and burnout are considerable.

## Conclusions

The present study showed a high prevalence of burnout among medical students in a private school using an active teaching methodology. In the first years of graduation, students’ personal attributes (optimism and self-perception of health) and school attributes (motivation and routine of the exhaustive study) were associated with higher levels of burnout. These results highlight the fact that burnout syndrome has multiple etiologies, with great involvement mainly in the first year. These findings reinforce the need to establish preventive measures focused on the personal attributes of first-year students, providing better performance, motivation, optimism, and empathy in the subsequent stages of the course.

## Supporting information

S1 Table Prevalence of burnout syndrome according with the three-dimensional criteria among medical students (n = 265). (DOC)

S2 Table Association between three-dimensional burnout scores and undergraduate school year (n = 265). (DOC)

S3 Table Association between three-dimensional burnout scores and the variables of the personal, school, and outside-of-school domains of medical students (n = 265). (DOC)

S4 Table Multiple logistic regression model of the three-dimensional criterion for burnout adjusted for the year of undergraduate school and the personal attributes of medical students (n = 265). (DOC)

S1 File Study database. (XLS)
