# Supplementary material for: Burnout among medical students during the first years of undergraduate school: Prevalence and associated factors
Source: PLoS One. 2018 Mar 7;13(3):e0191746. doi: 10.1371/journal.pone.0191746 (PMC5841647; doi:10.1371/journal.pone.0191746)
Supplement: S1 Table — (DOC) [file pone.0191746.s001.doc]

**S1 Table.** Prevalence of burnout syndrome according with the three-dimensional criteria among medical students (n = 265).

| **Three-Dimensional Burnout ^1^** | **N (%)** |
| --- | --- |
| No | 195 (73.6) |
| Yes | 70 (26.4) |

**Caption: ¹** High emotional exhaustion + high cynicism + low scores on the professional efficacy subscale; **RV =** Reference values.
