# Supplementary material for: Burnout among medical students during the first years of undergraduate school: Prevalence and associated factors
Source: PLoS One. 2018 Mar 7;13(3):e0191746. doi: 10.1371/journal.pone.0191746 (PMC5841647; doi:10.1371/journal.pone.0191746)
Supplement: S2 Table — (DOC) [file pone.0191746.s002.doc]

**S2 Table.** Association between three-dimensional burnout scores and undergraduate school year (Barretos School of Health Sciences, Dr. Paulo Prata, 2015-2016) (n = 265).

| **Variables** | ***Three-dimensional burnout*** | | |
| --- | --- | --- | --- |
|  | **No**  **N (%)** | **Yes N (%)** | **p*** |
| ***Undergraduate school year*** |  |  | < 0.001 |
| First Year | 66**^a^** (58.4) | 47**^b^** (41.6) |  |
| Second Year | 46**^a^** (82.1) | 10**^a^** (17.9) |  |
| Third Year | 44**^a^** (89.8) | 5**^b^** (10.2) |  |
| Fourth Year | 39**^a^** (83.0) | 8**^a^** (17.0) |  |

**Subtitle:** *****p-value *X* *^2^* test with linear tendency; each letter (a and b) denotes a subset of two-dimensional and three-dimensional categories of burnout with column proportions that do not differ significantly from each other.
