# Supplementary material for: Burnout among medical students during the first years of undergraduate school: Prevalence and associated factors
Source: PLoS One. 2018 Mar 7;13(3):e0191746. doi: 10.1371/journal.pone.0191746 (PMC5841647; doi:10.1371/journal.pone.0191746)
Supplement: S3 Table — (DOC) [file pone.0191746.s003.doc]

**S3 Table.** Association between three-dimensional burnout scores and the variables of the personal, school, and outside-of-school domains of medical students (Barretos School of Health Sciences, Dr. Paulo Prata, 2015-2016) (n=265).

| **Variables** | **Univariate**  **Three-Dimensional Burnout** | |
| --- | --- | --- |
|  | **With Burnout**  **N (%)** | **p*** |
| ***Personal Domain*** |  |  |
| ***Gender*** |  | 0.713 |
| Female | 48 (68.6) |  |
| Male | 22 (31.4) |  |
| ***Family income (1 *)*** |  | 0.469 |
| ≤ $3,000.00 | 38 (54.3) |  |
| ≥ $3,000.00 | 32 (45.7) |  |
| ***Self-perception of health*** |  | **< 0.001** |
| Bad | 40 (57.1) |  |
| Good | 30 (42.9) |  |
| ***Any health problem*** |  | 0.234 |
| Yes | 20 (28.6) |  |
| No | 50 (71.4) |  |
| ***Optimism*** |  | 0.136 |
| Not optimistic | 47 (67.1) |  |
| Optimistic | 23 (32.9) |  |
| ***Financial satisfaction*** |  | 0.368 |
| Dissatisfied | 23 (32.9) |  |
| Satisfied | 47 (67.1) |  |
| ***Fulfillment as a medical student*** |  | **< 0.001** |
| No | 29 (41.4) |  |
| Yes | 41 (58.6) |  |
| ***School Domain*** |  |  |
| ***School activities*** |  | 0.051 |
| Undergraduate school + other activity | 51 (72.9) |  |
| Undergraduate student only | 19 (27.1) |  |
| ***Average hours in college/day*** |  | **0.036** |
| Up to 12 hours | 59 (84.3) |  |
| More than 12 hours | 11 (15.7) |  |
| ***Feeling worn out or dissatisfaction as a student*** |  | 0.290 |
| Yes | 69 (98.6) |  |
| No | 1 (1.4) |  |
| ***Motivation for studies*** |  | **< 0.001** |
| No | 36 (67.9) |  |
| Yes | 17 (32.1) |  |
| ***Routine of studies*** |  | 0.338 |
| Exhaustive | 50 (71.4) |  |
| Not exhaustive | 20 (28.6) |  |
| ***Consider yourself important to colleagues*** |  | **0.030** |
| No | 32 (45.7) |  |
| Yes | 38 (54.3) |  |
| ***Satisfaction as a student*** |  | **< 0.001** |
| Dissatisfied | 47 (67.1) |  |
| Satisfied | 23 (32.9) |  |
| ***Outside-of-school Domain*** |  |  |
| ***Frequent family meetings*** |  | **0.028** |
| No | 50 (71.4) |  |
| Yes | 20 (28.6) |  |
| ***Leisure time*** |  | 0.109 |
| No | 64 (91.4) |  |
| Yes | 6 (8.6) |  |
| ***Has religion*** |  | **0.037** |
| No | 20 (28.6) |  |
| Yes | 50 (71.4) |  |
| ***Influence of religious/spiritual life in studies*** |  | **0.023** |
| No | 52 (74.3) |  |
| Yes | 18 (25.7) |  |
| ***Physical activity*** |  | **0.017** |
| No | 29 (41.4) |  |
| Yes | 41 (58.6) |  |
| ***Consider yourself important to your family members*** |  | 0.949 |
| No | 1 (1.4) |  |
| Yes | 69 (98.6) |  |
