# Supplementary material for: Burnout among medical students during the first years of undergraduate school: Prevalence and associated factors
Source: PLoS One. 2018 Mar 7;13(3):e0191746. doi: 10.1371/journal.pone.0191746 (PMC5841647; doi:10.1371/journal.pone.0191746)
Supplement: S4 Table — (DOC) [file pone.0191746.s004.doc]

**S4 Table. Multiple logistic regression model of the three-dimensional criterion for burnout adjusted for the year of undergraduate school and the personal attributes of medical students (n = 265).**

| **Variables** | **Model 1** | | **Model 2** | |
| --- | --- | --- | --- | --- |
|  | **OR (95% CI)** | **p** | **OR (95% CI)** | **p** |
| **Block 1 - Undergraduate school year** |  |  |  |  |
| First year | Ref. 1.0 | - | Ref. 1.0 | - |
| Second year | 0.3 (0.1-0.6) | **0.003** | 0.3 (0.1-0.8) | **0.025** |
| Third year | 0.1 (0.05-0.4) | **< 0.001** | 0.2 (0.07-0.6) | **0.004** |
| Fourth year | 0.2 (0.1-0.8) | 0.075 | 0.3 (0.1-0.8) | **0.020** |
| **Block 2 – Personal attribute** |  |  |  |  |
| Self-perception of health (good/bad) |  |  | 0.3 (0.1-0.6) | **0.001** |
| Fulfilled as student (yes/no) |  |  | 0.2 (0.1-0.5) | **< 0.001** |
| **R^2^ adjusted** | **0.131** | | **0.275** | |
| **Change in R^2^** |  | | **0.144** | |

**Legend: OR =** Odds ratio; ***** **P*-*value** obtained by Multiple logistic regression.
